# Expression, Characterization and Its Deinking Potential of a Thermostable Xylanase From *Planomicrobium glaciei* CHR43

**DOI:** 10.3389/fbioe.2021.618979

**Published:** 2021-02-17

**Authors:** Zhaoxing Liu, Tingting Shao, Yan Li, Bin Wu, Honghua Jia, Ning Hao

**Affiliations:** College of Biotechnology and Pharmaceutical Engineering, Nanjing Tech University, Nanjing, China

**Keywords:** endo-xylanase, thermo-alkali-stable, thermostable, psychrotolerant bacterium, enzymatic deinking

## Abstract

Genome mining is more and more widely used in identifying new enzymes from database. In the present study, we reported a putative xylanase, Pg-Xyn (WP_053166147.1), which originated from a psychrotolerant strain *Planomicrobium glaciei* CHR 43, and was identified from Genbank by genome mining. Sequence analysis and homology modeling showed that Pg-Xyn belongs to glycosyl hydrolase family 10. On the basis of heterologous expression in *E. coli* and biochemical characterization, we found Pg-Xyn was most active at pH 9.0 and 80°C and exhibited good stability from pH 5.0 to 12.0 and below 90°C. Pg-Xyn was slightly activated in the presence of Ca^2+^ and Mg^2+^, while it was strongly inhibited by Mn^2+^. The analysis of hydrolysis products showed that Pg-Xyn was an endo-β-1,4-xylanase. In addition, Pg-Xyn performed good deinking ability in a paper deinking test. In consideration of its unique properties, Pg-Xyn might be a promising candidate for application in the paper and pulp industries.

## Introduction

Xylanase (EC 3.2.1.8) catalyzes the hydrolysis of β-1,4-glycosidic linkages within xylan, playing the most important role in complete breakdown of xylan (Collins et al., 2005). In nature, xylanases exist in a variety of animals, plants, and microorganisms (Uday et al., 2018), and they are widely used in food, textile, ethanol production, waste treatment, and pulp and paper industries (Kumar et al., 2016; Kumar P. S. et al., 2018).

In the past years, much effort have been made to isolate xylanase producers, and a variety of microorganisms including bacteria, yeasts, and filamentous fungi have been found as xylanase producers, among which fungi have the ability to secrete xylanases with much higher yield than bacteria or yeast (Polizeli et al., 2005; Ahmed et al., 2009; Lombard et al., 2014). However, xylanases from bacterial sources show higher thermal stability than those from fungi (Mamo et al., 2006; Shrinivas et al., 2010; Zhang et al., 2012; Mhiri et al., 2020).

Nowadays, more and more genomic sequences have been deposited in the sequence databases, which afford huge treasure for mining xylanase candidates. *Planomicrobium glaciei* CHR43 is a psychrotolerant bacterium isolated from the cold desert region (Salwan et al., 2014), and its genome sequence was reported in 2014 (GenBank accession number: AUYR00000000.1). Until now, no record of a xylanase originating from *P. glaciei* CHR43 has been identified. Since the mesophilic and extremophilic bacteria are generally considered to be potent producers of industrial xylanases, when we used the sequence O30700 of a highly thermostable endo-xylanase isolated from mesophilic, alkalophilic *Bacillus* sp. NG-27 as a template that is most active at 70°C and pH 8.4 (Gupta et al., 2000) to run blastp job against UniParc database, a putative xylanase sequence from *P. glaciei* CHR43 (WP_053166147.1) was found to rank in low E-values. Considering that the selected sequence is highly identical to a xylanase from *Bacillus* sp. NG-27, we wonder whether the putative xylanase mined from the psychrotolerant bacterium would show similar properties. In the present study, the putative xylanase sequence from *P. glaciei* CHR43 was identified, heterologous expressed, and characterized, and its potential use for enzymatic deinking was also evaluated preliminarily.

## Materials and Methods

### Strains, Culture, and Chemicals

*E. coli* BL21(DE3), used as a host for the heterologous expression of xylanase, was purchased from TransGen Biotech (Beijing, China). Luria–Bertani (LB) medium was used to cultivate *E. coli* BL21(DE3) at 37°C. High-affinity Ni-NTA resins (L00250-C) were bought from GenScript (Nanjing, China). Xylans from bagasse, beechwood, birchwood, corncob and oat-spelt, as well as sodium carboxymethyl cellulose (CMC) and Avicel, were purchased from Sigma–Aldrich (St. Louis, MO, USA). All other chemicals were of analytical grade and commercially available.

### Gene Screening and Plasmid Synthesis

The sequence *Pg-Xyn* (WP_053166147.1) was aligned by BLAST search from the National Center for Biotechnology Information database using the xylanase sequence of *Bacillus* sp. NG-27 (O30700) as a template (Gupta et al., 2000) and a phylogenetic tree as reference. Plasmid was generated by inserting the potent xylanase gene, which was synthesized by GenScript (Nanjing, China), into the *Bam*HI-*Xho*I sites of pETDuet-1, which results in the addition of histidine tags to Pg-Xyn at N-terminus. The plasmid pETDuet-*Pg-Xyn* was transformed into *E. coli* BL21 (DE3), and their construction was confirmed by sequencing.

### Sequence and Putative Structure Analysis

Protein sequence similarity was evaluated using the BLASTp program (Altschul et al., 1990). Molecular mass was predicted using ProtParam tool (Gasteiger et al., 2005). Multiple sequence alignment was conducted with ClustalW (Thompson et al., 1994). Phylogenetic tree construction was performed using MEGA5.1 software according to the neighbor-joining algorithm (Tamura et al., 2007). Structural model was developed through homology modeling using SWISS-MODEL (Biasini et al., 2014). The crystal structure of a xylanase from *Bacillus* sp. NG-27 (PDB accession code 2FGL) was used as the template for Pg-Xyn. The visualization of the constructed model structure and the generation of graphical figure was performed with the PyMol molecular graphics system (http://www.pymol.org).

### Expression and Purification of Pg-Xyn

Plasmid pETDuet-1-*Pg-Xyn* was transformed into *E. coli* BL21(DE3) competent cells, and pETDuet-1 served as a control. Positive transformants harboring pETDuet-1-*Pg-Xyn* were harvested in 5 mL of LB medium containing 100 μg ml^−1^ ampicillin and grown overnight at 37 °C. Then, the overnight culture was shifted into 500 mL of LB medium containing ampicillin (100 μg mL^−1^) until the absorbance at 600 nm (A_600_) was 0.6. To express Pg-Xyn, isopropyl-β-D-1-thiogalactopyranoside (IPTG) was added to a final concentration of 1 mM, and the cultures were incubated for 12 h with vigorous agitation at 30°C. Then, the cultures were centrifuged at 10,000 × *g* at 4°C for 15 min. The cells were washed twice with ice-cold phosphate-buffered saline (PBS, 100 mM, pH 7.0), resuspended in the same buffer, and lysed by ultrasonication (220 W) in an ice bath. The lysate supernatant was obtained by centrifuge at 10,000 × *g* at 4°C for 20 min.

To purify the histidine-tagged recombinant Pg-Xyn, the clarified supernatant was incubated at 50°C for 30 min and then centrifuged at 10,000 × *g* at 4°C for 10 min to collect the supernatant. The supernatant was loaded onto a Ni-NTA column and eluted with a linear imidazole gradient (50–200 mM). The purity and apparent molecular mass of purified Pg-Xyn was determined using sodium dodecyl sulfate-polyacrylamide gel electrophoresis (SDS-PAGE). Protein concentration was measured via the Bradford method (Bradford, 1976).

### Enzyme Activity Assay

The determination of Pg-Xyn activity was based on the amount of reducing sugar produced from substrates, as determined by the 3,5-dinitrosalicylic acid (DNS) method (Miller, 1959). The mixture consisted of 0.2 mL of properly diluted enzyme and 1.8 mL of 1% (w/v) brichwood xylan (Sigma) in PBS buffer (pH 4.8), and it was incubated at 50 °C for 10 min, and then 2 mL of DNS was added to stop the reaction. The mixture was boiled for 10 min and the A_540_ was measured after cooling the mixture to room temperature. A solution with the same amount of heat-denatured Pg-Xyn and DNS was used as a control. All activity measurements were carried out in triplicate. One unit of enzyme activity was defined as the amount of enzyme necessary to produce 1 μmol of reducing sugar per minute.

### Optimal pH and pH Stability

The optimal pH of Pg-Xyn was investigated by measuring its activity at 37°C for 10 min using the following buffer solutions between pH 3 and 12. Buffers were citrate-Na_2_HPO_4_ (pH 3–8), glycine-NaOH (pH 9–10), and Na_2_HPO_4_-NaOH (pH 11–12). To examine their pH stabilities, Pg-Xyn was added into various buffers ranging from pH 3 to 12, and incubated with brichwood xylan at 37°C for 12 h.

### Optimal Temperature and Thermal Stability

The optimal temperature for Pg-Xyn activity was determined from 30 to 90°C at the optimal pH. Enzyme thermostability was determined at temperatures ranging from 50 to 90°C for 1, 2, 3, 4, 5, and 6 h at the optimal pH by measuring the residual activity.

### Effect of Metal Ions and Chemical Reagents

To determine the effect of various metal ions and chemical reagents on Pg-Xyn activity, metal ions (Cu^2+^, Zn^2+^, Fe^2+^, Fe^3+^, Ba^2+^, Mg^2+^, Mn^2+^, and Ca^2+^) and chemical reagents (EDTA and SDS) were mixed with the purified Pg-Xyn at 1 mM. Pg-Xyn activity was evaluated under the optimal pH and temperature, and a reaction with no additive was used as a control.

### Kinetic Studies and Substrate Specificity

The kinetic constants of Pg-Xyn were determined by a Lineweaver–Burk plot. Reactions were conducted using the brichwood xylan as the substrate under the optimal conditions. To probe into its substrate specificity, Pg-Xyn was incubated with one of the following substrates (1%, w/v): xylans from beechwood, corncob, oat-spelt, bagasse, CMC-Na, starch, and Avicel. Pg-Xyn activity was also examined.

### Analysis of Xylan Hydrolysis Products

Reactions, including 10 mL of 1% (w/v) substrate (beechwood xylan, corncob xylan, oat-spelt xylan, or bagasse xylan) and 50 μg of the purified Pg-Xyn were incubated at 50°C for 24 h. The hydrolysis products were detected by HPLC using a Zorbax NH_2_ column (Agilent, USA) and a refractive index detector. The mobile phase consisted of 80% acetonitrile and 20% water, and the flow rate was 1.0 ml min^−1^. Xylose, xylobiose, xylotriose, xylotetraose, and xylohexaose were used as the standards.

### Deinking of Laser-Printed Paper Using Pg-Xyn

A printed copy page (A4 size) from a Hewlett-Packard laser printer was used for deinking. The paper was printed with black ink. A fixed amount (100 mg) of cut paper (3 × 3 cm) was stripped. The paper strip was dipped into phosphate buffer (pH 7.0) containing Pg-Xyn (1 U mg^−1^) and incubated overnight at the optimal temperature. A control with heat-denatured Pg-Xyn was also used. The deinking of the laser-printed paper was measured by testing the absorbance of the released color in the filtrate at 596 nm. All experiments were conducted in triplicate. Enzymatically treated pulp was further observed by microscopy, and laser paper without enzymatic treatment was used as a control.

## Results

### Sequence Analysis of Pg-Xyn

**Figure 1:**
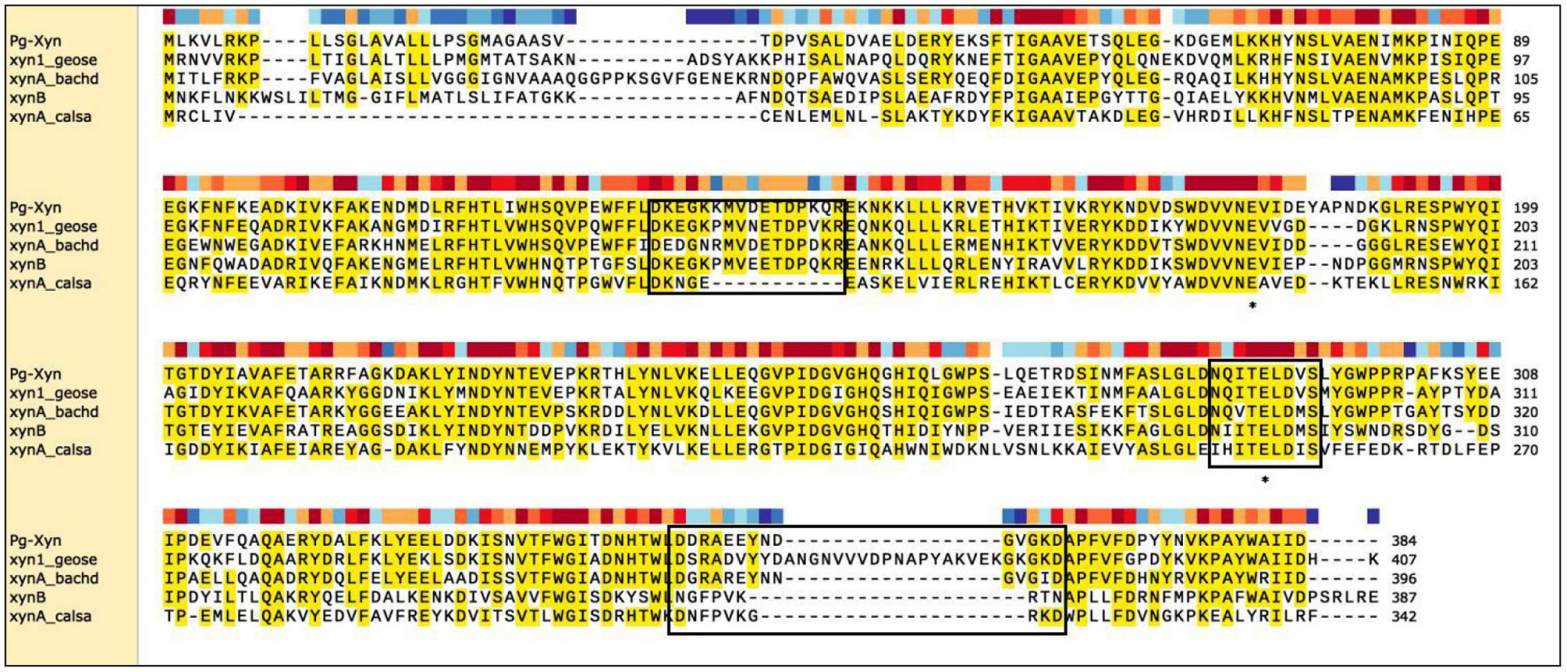
Sequence comparison of Pg-Xyn to the other xylanases. Amino acid sequence identical to at least four of the aligned sequences are shaded in yellow, this area is highly conservative. Three boxed regions stretches that are unique to alkalophilic xylanases. The residues marked with “*” are the putative active site in family 10 xylanase. Sequence name, microbial source and UniProt Kowledgebase accession number were given as follows: Pg-Xyn *Planomicrobium glaciei* CHR43 (WP_053166147.1); xyn1_geose *Geobacillus stearothermophilus* (P40943); xynA_bachd *Bacillus halodurans* (P07528); xynB *Clostridium stercorarium* (P40942); xynA_calsa *Caldicellulosiruptor saccharolyticus* (P23556).

The complete coding sequence for Pg-Xyn from *P. glaciei* CHR43 was 1,152 bp. The calculated molecular mass of Pg-Xyn is 44.06 kDa. Sequence analysis showed that Pg-Xyn, belongs to GH10, showing 43–65% identity with those xylanases from *Geobacillus stearothermophilus* (P40943), *B. halodurans* (P07528), and *Clostridium stercorarium* (P40942), *Caldicellulosiruptor saccharolyticus* (P23556) using UniProt Knowledgebase searches. Pg-Xyn has two catalytic residues, Glu (179) and Glu (289) according to the sequence alignment of Pg-Xyn with the other 4 family 10 xylanases (Figure 1).

**Figure 2:**
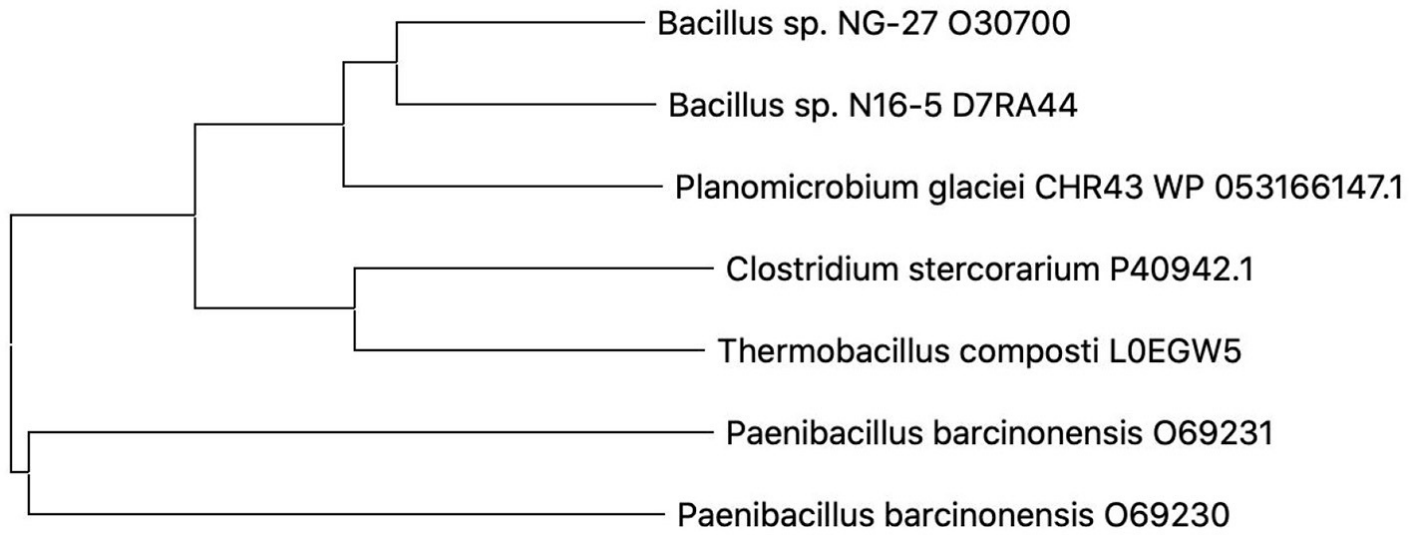
Phylogenetic tree constructed using the neighbor-joining method (MEGA 5.1). Microbial source and UniProt Bnowledgebase accession number for the selected protein sequences were given as follows: *Bacillus* sp. NG-27 (O30700), Bacillus sp. N16-5 (D7RA44), *Planomicrobium glaciei* CHR43 (WP_053166147.1), *Clostridium stercorarium* (P40942), *Thermobacillus composti* (L0EGW5), *Paenibacillus barcinonensis* (O69230), and *Paenibacillus barcinonensis* (O69231).

A phylogenetic tree was built on the basis of the amino acid sequence of Pg-Xyn (Figure 2). The closest homolog for Pg-Xyn is xylanase from *Bacillus* sp. NG-27 (O30700), indicating that it could have higher structural similarity. The higher structural similarity can endow Pg-Xyn similar enzymatic properties with thermostable xylanases from some *Bacillus* genera.

**Figure 3:**
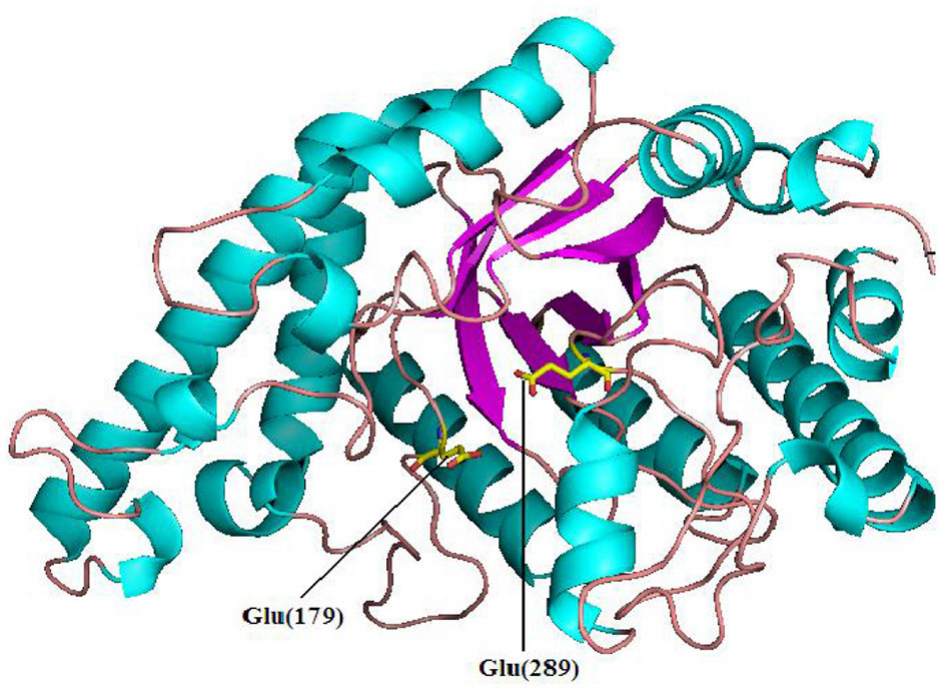
Structure prediction of Pg-Xyn by SWISS-Model. Glu 179 and Glu 289 are marked.

Pg-Xyn has 64.2% sequence identity with xylanase O30700 from *Bacillus* sp. NG-27. After Swiss-model modeling, it is found that although the detailed interaction sites between Pg-Xyn and Xylan are different, the superposition shows that their characteristic motifs and corresponding active regions are highly similar in structure, and the active pocket regions are quite conservative. Homology modeling revealed that Pg-Xyn has the same 8-fold (β/α) structure as thermostable F/10 xylanase O30700 (PDB 2FGL). This 8-fold (β/α) or TIM barrel fold typically found in all GH10 xylanases. Pg-Xyn and 2FGL have similar conserved sites Asp and Glu, which are conserved in alkalophilic xylanases, but less conserved in non-alkalo-philic xylanases (Manikandan et al., 2006). On the basis of the 3 D-structure of Pg-Xyn, two glutamic acid residues, Glu 179 and Glu 289, were identified as putative catalytic residues (Figure 3).

### Expression and Purification of Pg-Xyn

**Figure 4:**
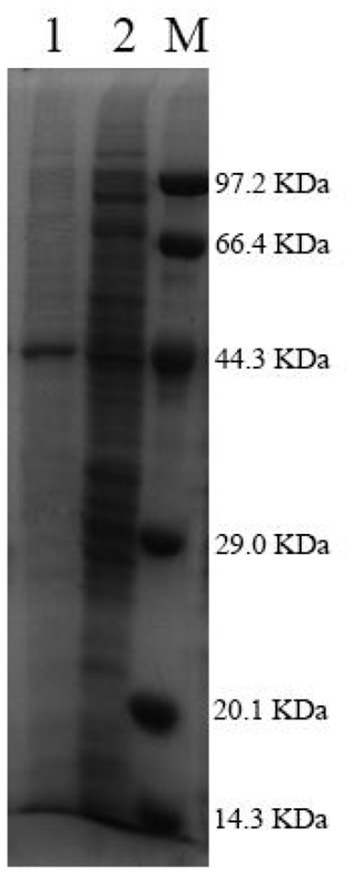
SDS-PAGE of the purified Pg-Xyn. M, Low protein marker; 1, Pg-Xyn purified enzyme; 2, Pg-Xyn crude enzyme.

After being expressed in *E. coli* BL21 (DE3), Pg-Xyn showed the highest specific activity of 1.08 × 10^4^ U mg^−1^ in crude cell extracts. Pg-Xyn was treated at 50°C for 30 min and then purified by Ni-NTA column chromatography. Pg-Xyn exhibited a yield of 83% after purification from the crude extract. The specific activity of the purified Pg-Xyn reached 1.95 × 10^4^ U mg^−1^. The purified Pg-Xyn was analyzed by SDS-PAGE. There was a single band of ~44.3 kDa, which agreed with its theoretical molecular weight (Table 1; Figure 4).

**Table 1:** Summary of the purification of Pg-Xyn.

**Step**	**Total protein (mg)**	**Total activity (U)**	**Specific activity (U mg^**−1**^)**	**Purification (fold)**	**Yield (%)**
Crude extraction	163	1.76 × 10^6^	1.08 × 10^4^	1	100
Ni-NTA	75	1.46 × 10^6^	1.95 × 10^4^	2.17	83

### Biochemical Characterization of the Purified Pg-Xyn

**Figure 5:**
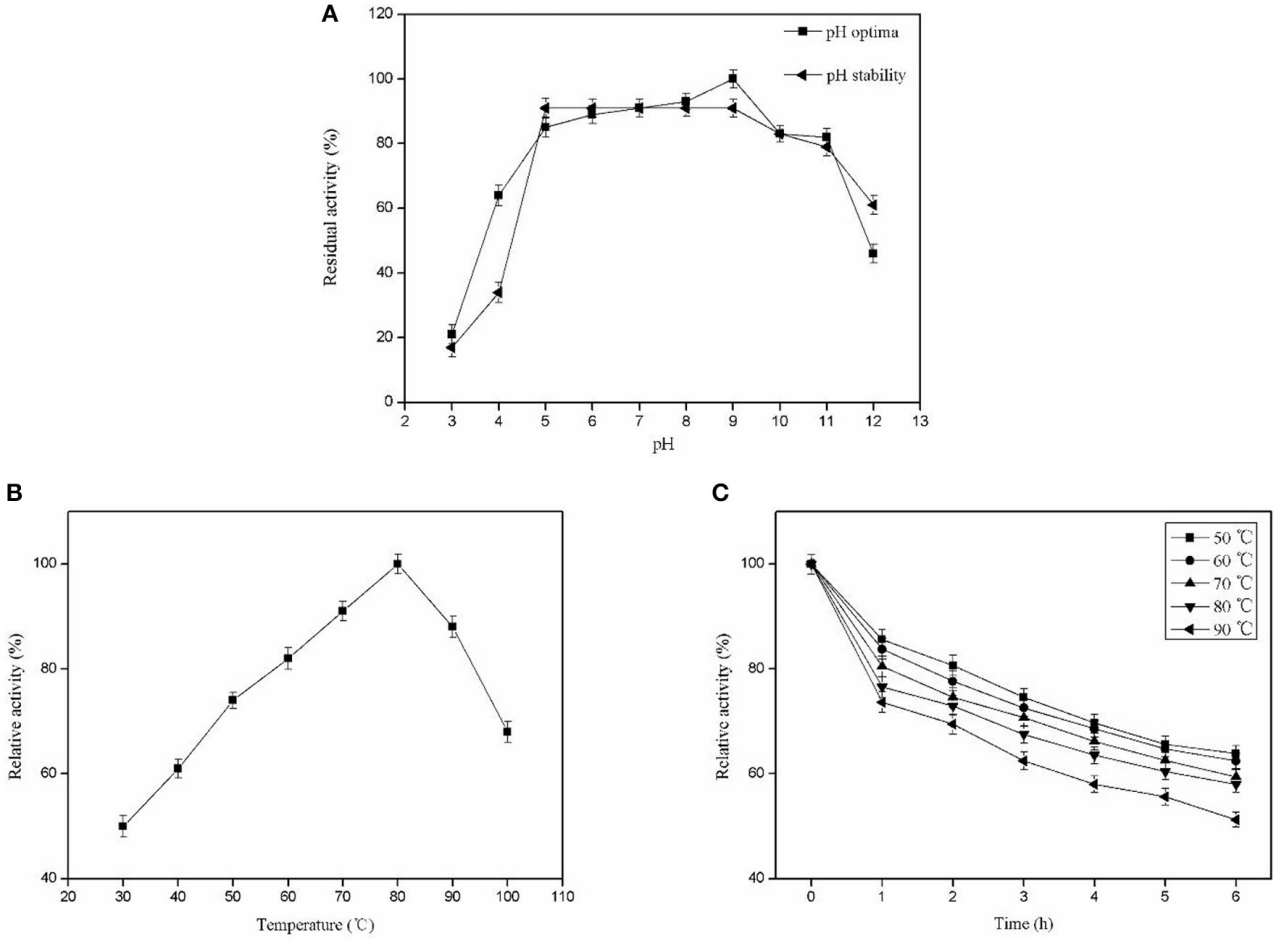
Biochemical characterization of the purified Pg-Xyn. **(A)** Effect of pH on Pg-Xyn activity and pH stability of Pg-Xyn; **(B)** Effect of temperature on Pg-Xyn activity; **(C)** Thermal stability of Pg-Xyn.

The optimal pH for Pg-Xyn was pH 9, presenting high activity between pH 5 and 11. After incubation at 37°C for 12 h, Pg-Xyn retained more than 80% of its activity (Figure 5A). The pH characteristic of Pg-Xyn was similar to that of a xylanase from *Bacillus* sp. NG-27 (Gupta et al., 2000). The optimal temperature for Pg-Xyn was 80°C at pH 9.0 (Figure 5B). Pg-Xyn retained >50% of its activity between 50 and 90°C after 6 h (Figure 5C). The optimal temperature for Pg-Xyn was higher than those of earlier reports for most bacterial xylanases, as the optimal temperatures of *Bacillus* sp. PKD-9, *Bacillus arsenici*s*elenatis* DSM 15340, *Bacillus pumilus*, and *Paenibacillus campinasensis* G1-1 range from 50 to 60°C (Kamble and Jadhav, 2012; Zhang et al., 2012; Thomas et al., 2014). These results suggested that Pg-Xyn is an alkali-thermostable xylanase.

The purified Pg-Xyn activity was tested in the presence of metal ions and chemical agents (Table 2). Pg-Xyn was slightly activated in the presence of Ca^2+^ and Mg^2+^, while it was inhibited by the addition of Cu^2+^, Fe^2+^, Fe^3+^, and EDTA. In particular, Pg-Xyn was strongly inactivated by Mn^2+^. These results were similar to the other xylanases in previous publications (Ratanakhanokchai et al., 1999; Ping et al., 2018).

**Table 2:** Effect of metal ions and chemical reagents on the activity of the purified Pg-Xyn.

**Relative activity (%)**	**Pg-Xyn**
Control	100
Cu^2+^	91.3
Zn^2+^	101
Fe^3+^	88.0
Fe^2+^	88.4
Ba^2+^	91.3
Mg^2+^	104
Mn^2+^	58.0
Ca^2+^	105
EDTA	86.7
SDS	98.0

### Kinetic Analysis

**Figure 6:**
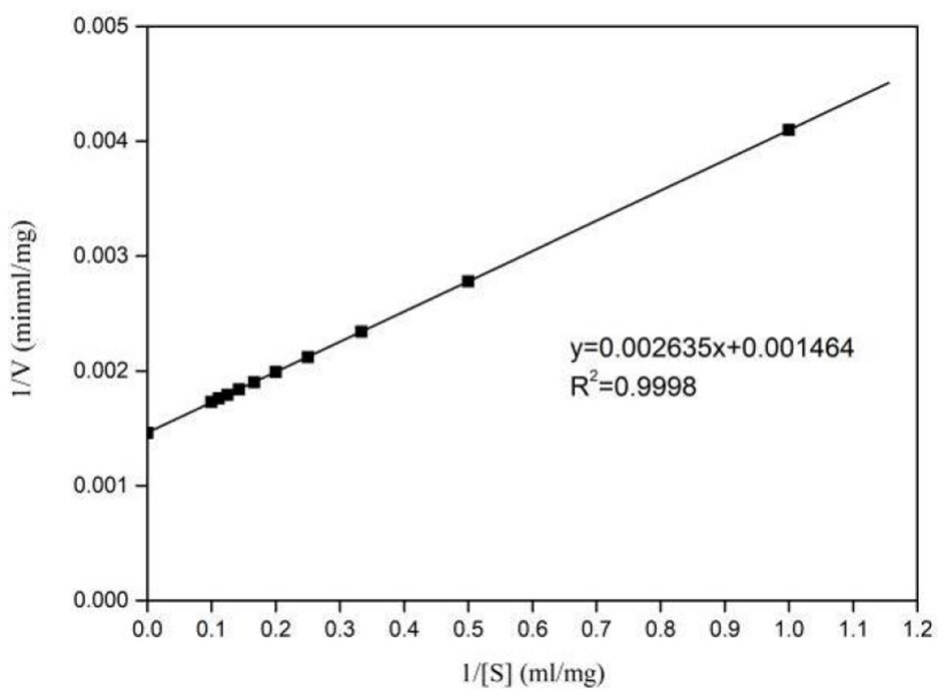
Lineweaver-Burk plots of Pg-Xyn kinetic analysis.

Kinetic parameters of Pg-Xyn were determined according to Lineweaver–Burk plots (Figure 6), and the *K*_m_ and *V*_max_ values of the purified Pg-Xyn were 1.8 mg mL^−1^ and 683 U mg^−1^, respectively.

The catalytic constant (*k*_cat_/*K*_m_) can be used to compare the catalytic ability of enzymes (Zhao et al., 2013; Table 3). Compared with other bacterial xylanases, the *k*_cat_/*K*_m_ of Pg-Xyn was 318.9 mL mg^−1^ s^−1^, which is higher than that of other bacterial xylanases (Qiu et al., 2010; Verma and Satyanarayana, 2012; Ribeiro et al., 2014; An et al., 2015).

**Table 3:** Comparison of the enzymatic properties of Pg-Xyn with those of other bacterial xylanases.

**Xylanase**	**Organism**		**Optimum**	**V_**max**_**	**K_**m**_**	***k*_**cat**_**	***k*_**cat**_/K_**m**_**	**References**
		**pH**	**Temperature (**°**C)**	**(U mg^**−1**^)**	**(mg mL^**−1**^)**	**(s^**−1**^)**	**(mL mg^**−1**^ s^**−1**^)**	
MpXyn10A	*Malbrancheapulchella*	5.8	80	82	4.6	748	162	Ribeiro et al., 2014
Xyn10B	*Caldicellulosiruptorbescii*	7.2	70	450	2.16	321.6	148.8	An et al., 2015
XYNAM6	*Streptomyces megasporus*	5.5	70	322.69	2.33	255.89	109.82	Qiu et al., 2010
xyl-gt	*Geobacillusthermoleovorans*	8.5	80	42.5	2.1	318.75	151.78	Verma and Satyanarayana, 2012
Pg-Xyn	*P. glaciei*CHR43	9.0	80	683	1.8	574	318.9	This study

### Substrate Specificity

The substrate specificity of the purified Pg-Xyn is displayed in Table 4. The purified Pg-Xyn can hydrolyze corncob xylan effectively, and it showed slightly lower activity on oat-spelt and birchwood xylans, while it exhibited lower activity on bagasse and beechwood xylans. No activity was detected on CMC-Na, starch, and Avicel, indicating that Pg-Xyn is a cellulase-free xylanase.

**Table 4:** Substrate specificity of Pg-Xyn.

**Substrate**	**Concentration (%)**	**Relative activity (%)**
Corncob xylan	1	100
Oat-spelt xylan	1	78.1
Bagasse xylan	1	31.2
Beechwood xylan	1	40.6
Birchwood xylan	1	78.1
CMC-Na	1	0
Avicel	1	0

### Analysis of Corncob Xylan Hydrolysis Products

Pg-Xyn has the highest specificity activity to corncob xylan, so we chose corncob xylan as the substrate of the hydrolysis reaction for HPLC analysis. The products of corncob xylan hydrolyzed with the purified Pg-Xyn were determined by HPLC analysis of the reaction solution (Table 5). The major products are xylose, xylobiose and xylotriose, but no xylotetraose was observed (Supplemental Figure 1). According to these results, we confirm that Pg-Xyn is an endo-β-1,4-xylanase.

**Table 5:** Hydrolysis products of corncob xylan by Pg-Xyn.

**Product**	**Xylose**	**Xylobiose**	**Xylotriose**	**Xylotetraose**
Percentage (%)	48.07 ± 0.03	36.89 ± 0.02	15.04 ± 0.02	0

### Enzymatic Deinking

**Figure 7:**
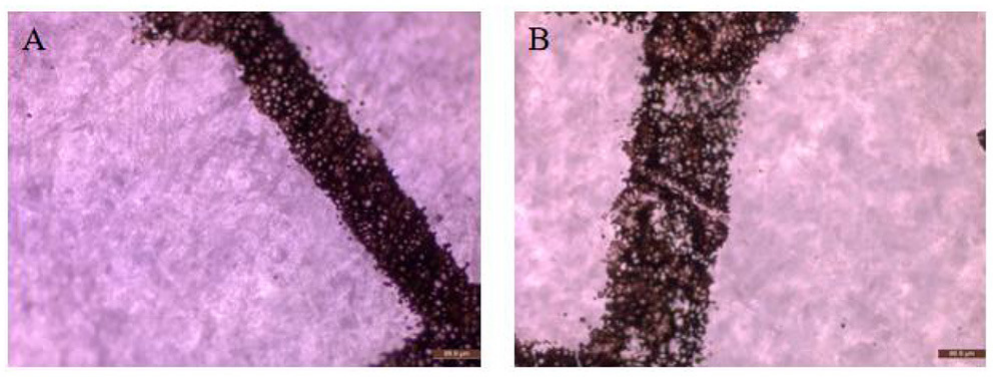
Microscopic view of laser printed paper. **(A)** Control; **(B)** Treated by Pg-Xyn.

Xylanase can increase the relative hydrophobicity of the surface of paper, and facilitate the detachment of adsorbed ink particles. The evaluation of the paper deinking by the newly characterized xylanase revealed that Pg-Xyn had 298% deinking activity, which is higher than 239.58% by a purified xylanase from *Bacillus* sp. CKBx1D (Maity et al., 2012). The effect of Pg-Xyn on deinking was further investigated by microscopy, as is showed in Figure 7. The results clearly show that the paper lost its fiber compactness and became brighter after enzymatic treatment.

## Discussion

Psychrophiles and psychrotolerants are found widely in nature, producing a lot of enzymes to be cold active and general thermolabile. However, thermostable enzymes deriving from psychrophilesare also not uncommon. For example, a thermostable xylose isomerase originating from the psychrophilic soil microorganism, *Paenibacillus* sp. appears to be most active at 60°C (Park et al., 2019). Oikawa et al. found an aldehyde dehydrogenase and an aspartase with the highest activity at 55°C deriving from *Cytophaga* sp. KUC-1 (Oikawa et al., 2003). The recombinant L-haloacid dehalogenase isolated from a marine bacterium *Psychromonas ingrahamii* is most active at 45°C and retains above 70% of its activity after being incubated at 65°C for 90 min (Novak et al., 2013). In the present study, we mined a thermophilic xylanase Pg-Xyn (WP_053166147.1) from the genome sequence of a psychrotolerant bacteria *P. glaciei* CHR43. The deduced amino acid sequence for Pg-Xyn has strong similarity to some other family 10 xylanases from bacteria. Biochemical characterization showed that Pg-Xyn was thermostable and alkaline preference with the highest activity at pH 9.0 and 80°C. According to previous publications, similar xylanases active at elevated temperatures and alkaline pH have ever been reported. For instance, XynDZ5, a newly reported thermostable xylanolytic enzyme derived from *Thermoanaerobacterium thermosaccharolyticum*, performs best at 65–75°C and pH 7.5 (Zarafeta et al., 2020). Another xylanase XynHB originating from *B. pumilus* HBP8 was extracellular secreted expressed in *E. coli*, which have showed high activity under alkaline conditions at 70°C (Zhang et al., 2020). Additionally, a novel xylanse identified from an extreme temperature hot spring metagenome was most active at pH 7 and 80°C and exhibited significant pH stability at pH ranging from 6 to 9 (Joshi et al., 2020).

Xylanases are distributed among two GH families, family 10 and family 11, according to hydrophobic cluster analysis and sequence alignment (Henrissat et al., 1991; Davies and Henrissat, 1995). Most alkali-thermostable xylanases belonging to GH 10 were isolated from alkalophilic, thermophilic or mesophilic bacteria, such as the genus *Paenibacillus, Geobacillus*, and *Bacillus* (Zhao et al., 2011; Canakci et al., 2012; Thomas et al., 2014). So far none of alkali-thermostable xylanases has been reported to be identified in the genus *Planomicrobium* inhabiting cold area. On the basis of the results, this study highlights a new resource for thermostable xylanase producers. In the light of the good thermal performance, Pg-Xyn could attract the interests from many industrial processes. The application of xylanases has long been recognized as an effective bleaching pretreatment in the industries seeking to reduce the use of chlorine-containing bleach agents, thereby minimizing the negative environmental impact (Woolridge, 2014).

In the recent years, deinking of paper pulp employing enzymes has attracted more and more attention. Of these enzymes, cellulase, xylanase, and laccase have usually been used in deinking of paper waste. Xylanase isozymes from *Bacillus* sp. CKBx1D and cellulase-xylanase complex from *E. coli* SD5 have been found to exhibit deinking activity and to enhance the brightness of waste pulp (Maity et al., 2012; Kumarab N. V. et al., 2018). According to deinking test, Pg-Xyn showed the higher activity in enzymatic deinking. In view of its good thermal stability and higher activity, we believe Pg-Xyn might be a good candidate to be utilized in pulp and paper industries.

## Data Availability Statement

The original contributions presented in the study are included in the article/Supplementary Material, further inquiries can be directed to the corresponding author/s.

## Author Contributions

HJ and NH conceived and designed the experiments. ZL and TS performed the experiments and analyzed the data. BW and YL contributed to experimental design and also critically revised the manuscript. ZL drafted the manuscript. All authors read and approved the final manuscript.

## Conflict of Interest

The authors declare that the research was conducted in the absence of any commercial or financial relationships that could be construed as a potential conflict of interest.

## References

[B1] Ahmed S. Riaz S. Jamil A. (2009). Molecular cloning of fungal xylanases: an overview. Appl. Microbiol. Biot. 84, 19–35. 10.1007/s00253-009-2079-4 19568746

[B2] Altschul S. F. Gish W. Miller W. Myers E. W. Lipman D. J. (1990). Basic local alignment search tool. J. Mol. Biol. 215, 403–410. 10.1016/S0022-2836(05)80360-2 2231712

[B3] An J. Xie Y. Zhang Y. Tian D. Wang S. Yang G.. (2015). Characterization of a thermostable, specific GH10 xylanase from *Caldicellulosiruptor bescii* with high catalytic activity. J. Mol. Catal. B Enzym. 117, 13–20. 10.1016/j.molcatb.2015.04.003

[B4] Biasini M. Bienert S. Waterhouse A. Arnold K. Studer G. Schmidt T.. (2014). SWISS-MODEL: modelling protein tertiary and quaternary structure using evolutionary information. Nucleic Acids Res. 42, 252–258. 10.1093/nar/gku340 24782522 PMC4086089

[B5] Bradford M. M. (1976). A rapid and sensitive method for the quantitation of microgram quantities of protein utilizing the principle of protein-dye binding. Anal. Biochem. 72, 248–254. 10.1016/0003-2697(76)90527-3 942051

[B6] Canakci S. Cevher Z. Inan K. Tokgoz M. Bahar F. Kacagan M.. (2012). Cloning, purification and characterization of an alkali-stable endoxylanase from thermophilic *Geobacillus* sp. 71. World J. Microbiol. Biotechnol. 28, 1981–1988. 10.1007/s11274-011-1000-3 22806019

[B7] Collins T. Gerday C. Feller G. (2005). Xylanases, xylanase families and extremophilic xylanases. FEMS Microbiol. Rev. 29, 3–23. 10.1016/j.femsre.2004.06.005 15652973

[B8] Davies G. Henrissat B. (1995). Structures and mechanisms of glycosyl hydrolases. Structure 3, 853–859. 10.1016/S0969-2126(01)00220-9 8535779

[B9] Gasteiger E. Hoogland C. Gattiker A. Duvaud S. Wilkins M. R. Appel R. D.. (2005). Protein identification and analysis tools on the ExPASy server, in The Proteomics Protocols Handbook, ed Walker J. M. (Totowa, NJ: Humana Press), 571–607.

[B10] Gupta N. Reddy V. S. Maiti S. Ghosh A. (2000). Cloning, expression, and sequence analysis of the gene encoding the mesophilic *Bacillus* sp. strain NG-27. Appl. Environ. Microbial. 66, 2631–2635. 10.1128/AEM.66.6.2631-2635.2000 10831448 PMC110591

[B11] Henrissat B. Vegetales M. Grenoble F. (1991). A classification of glycosyl hydrolases based sequence similarities amino acid. Biochem. J. 280, 309–316. 10.1042/bj2800309 1747104 PMC1130547

[B12] Joshi N. Sharma M. Singh S. P. (2020). Characterization of a novel xylanase from an extreme temperature hot spring metagenome for xylooligosaccharide production. Appl. Microbiol. Biotechnol. 104, 4889–4901. 10.1007/s00253-020-10562-7 32249395

[B13] Kamble R. D. Jadhav A. R. (2012). Isolation, purification, and characterization of xylanase produced by a new species of *Bacillus* in solid state fermentation. Int. J. Microbiol. 2012:683193. 10.1155/2012/683193 22315613 PMC3270423

[B14] Kumar P. S. Yaashikaa P. R. Saravanan A. (2018). Isolation, characterization and purification of xylanase producing bacteria from sea sediment. Biocatalysis and agricultural biotechnology 13, 299–303. 10.1016/j.bcab.2018.01.007

[B15] Kumar V. Marín-Navarro J. Shukla P. (2016). Thermostable microbial xylanases for pulp and paper industries: trends, applications and further perspectives. World J. Microbial. Biotechnol. 32:34. 10.1007/s11274-015-2005-0 26754672

[B16] Kumarab N. V. Rania M. E. Gunaseelic R. Kannand N. D. (2018). Paper pulp modification and deinking efficiency of cellulase-xylanase complex from *Escherichia coli* SD5. Int. J. Biol. Macromol. 111, 289–295. 10.1016/j.ijbiomac.2017.12.126 29309867

[B17] Lombard V. GolacondaRamulu H. Drula E. Coutinho P. M. Henrissat B. (2014). The carbohydrate-active enzymes database (CAZy) in 2013. Nucleic Acids Res. 42, 490–495. 10.1093/nar/gkt1178 24270786 PMC3965031

[B18] Maity C. Ghosh K. Halder S. K. Jana A. Adak A. Mohapatra P. K.. (2012). Xylanase isozymes from the newly isolated *bacillus* sp. CKBx1D and optimization of its deinking potentiality. Appl. Biochem. Biotechnol. 167, 1208–1219. 10.1007/s12010-012-9556-4 22278053

[B19] Mamo G. Hatti-Kaul R. Mattiasson B. (2006). A thermostable alkaline active endo-β-1-4-xylanase from *Bacillus halodurans* S7: purification and characterization. Enzyme Microb. Technol. 39, 1492–1498. 10.1016/j.enzmictec.2006.03.040

[B20] Manikandan K. Bhardwaj A. Gupta N. Lokanath N. K. Ghosh A. Reddy V. S.. (2006). Crystal structures of native and xylosaccharides-bound alkali thermostable xylanase from an alkalophilic *Bacillus* sp. NG-27: structural insights into alkalophilicity and implications for adaptation to polyextreme conditions. Protein Sci. 15, 1951–1960. 10.1110/ps.062220206 16823036 PMC2242578

[B21] Mhiri S. Bouanane-Darenfed A. Jemli S. Neifar S. Bejar S. (2020). A thermophilic and thermostable xylanase from *Caldicoprobacter algeriensis*: recombinant expression, characterization and application in paper biobleaching. Int. J. Biol. Macromol. 164, 808–817. 10.1016/j.ijbiomac.2020.07.162 32698070

[B22] Miller G. L. (1959). Use of dinitrosalicylicacid reagent for determination of reducing sugar. Anal. Chem. 31, 426–428. 10.1021/ac60147a030

[B23] Novak H. R. Sayer C. Panning J. Littlechild J. A. (2013). Characterisation of an L-haloacid dehalogenase from the marine psychrophile *Psychromona singrahamii* with potential industrial application. Marine Biotechnol. 15, 695–705. 10.1007/s10126-013-9522-3 23949008

[B24] Oikawa T. Kazuoka T. Soda K. (2003). Paradoxical thermostable enzymes from psychrophile: molecular characterization and potentiality for biotechnological application. J. Mol. Catal. B Enzym. 23, 65–70. 10.1016/S1381-1177(03)00073-0

[B25] Park S. H. Kwon S. Lee C. W. Kim C. M. Jeong C. S. Kim K. J.. (2019). Crystal structure and functional characterization of a xylose isomerase (PBXI) from the psychrophilic soil microorganism, *Paenibacillus* sp. J. Microbiol. Biotechnol. 29, 244–255. 10.4014/jmb.1810.10057 30602271

[B26] Ping L. F. Wang M. J. Yuan X. L. Cui F. J. Huang D. M. Sun W. J.. (2018). Production and characterization of a novel acidophilic and thermostable xylanase from *Thermoascus aurantiacu*. Int. J. Biol. Macromol. 109, 1270–1279. 10.1016/j.ijbiomac.2017.11.130 29175163

[B27] Polizeli M. L. T. M. Rizzatti A. C. S. Monti R. Terenzi H. F. Jorge J. A. Amorim D. S. (2005). Xylanases from fungi: properties and industrial applications. Appl. Microbiol. Biotechnol. 67, 577–591. 10.1007/s00253-005-1904-7 15944805

[B28] Qiu Z. Shi P. Luo H. Bai Y. Yuan T. Yang P.. (2010). A xylanase with broad pH and temperature adaptability from *Streptomyces megasporus* DSM 41476, and its potential application in brewing industry. Enzyme Microb. Technol. 46, 506–512. 10.1016/j.enzmictec.2010.02.003 25919627

[B29] Ratanakhanokchai K. Kyu K. L. Tanticharoen M. (1999). Purification and properties of a xylan-binding endoxylanase from alkaliphilic *Bacillus* sp. strain K-1. Appl. Environ. Microbiol. 65, 694–697. 10.1128/AEM.65.2.694-697.1999 9925602 PMC91081

[B30] Ribeiro L. F. C. De Lucas R. C. Vitcosque G. L. Ribeiro L. F. Ward R. J. Rubio M. V.. (2014). A novel thermostable xylanase GH10 from *Malbranchea pulchell*a expressed in *Aspergillus nidulans* with potential applications in biotechnology. Biotechnol. Biofuels 7:115. 10.1186/1754-6834-7-115 25788980 PMC4364333

[B31] Salwan R. Swarnkar K. Singh K. Kasana C. (2014). First draft genome sequence of a member of the genus *Planomicrobium*, isolated from the Chandra river, India. Genome Announc. 2, e01259–e01213. 10.1128/genomeA.01259-13 24503999 PMC3916493

[B32] Shrinivas D. Savitha G. Raviranjan K. Naik G. R. (2010). A highly thermostable alkaline cellulase-free xylanase from thermoalkalophilic*bacillus* sp. JB 99 suitable for paper and pulp industry: purification and characterization. Appl. Biochem. Biotechnol. 162, 2049–2057. 10.1007/s12010-010-8980-6 20467831

[B33] Tamura K. Dudley J. Nei M. Kumar S. (2007). MEGA4: molecular evolutionary genetics analysis (MEGA) software version 4.0. Mol. Biol. Evol. 24, 1596–1599. 10.1093/molbev/msm092 17488738

[B34] Thomas L. Ushasree M. V. Pandey A. (2014). An alkali-thermostable xylanase from *Bacillus pumilus* functionally expressed in *Kluyveromyces lactis* and evaluation of its deinking efficiency. Bioresour. Technol. 165, 309–313. 10.1016/j.biortech.2014.03.037 24709528

[B35] Thompson J. D. Higgins D. G. Gibson T. J. (1994). Clustal-W - improving the sensitivity of progressive multiple sequence alignment through sequence weighting, position-specific gap penalties and weight matrix choice. Nucleic Acids Res. 22, 4673–4680. 10.1093/nar/22.22.4673 7984417 PMC308517

[B36] Uday U. S. P. Goswami S. Gopikrishna K. Bandyopadhyay T. K. Bhunia B. (2018). Identification of markers at various stages of batch fermentation and improved production of xylanase using *Aspergillus niger* (KP874102.1). 3 Biotech 8:337. 10.1007/s13205-018-1363-3 30073122 PMC6057856

[B37] Verma D. Satyanarayana T. (2012). Cloning, expression and applicability of thermo-alkali-stable xylanase of *Geobacillus thermoleovorans* in generating xylooligosaccharides from agro-residues. Bioresour. Technol. 107, 333–338. 10.1016/j.biortech.2011.12.055 22212694

[B38] Woolridge E. M. (2014). Mixed enzyme systems for delignification of lignocellulosic biomass. Catalysts 4, 1–35. 10.3390/catal4010001

[B39] Zarafeta D. Galanopoulou A. P. Leni M. E. Kaili S. I. Chegkazi M. S. Chrysina E. D.. (2020). XynDZ5: a new thermostable GH10 xylanase. Front. Microbiol. 11:545. 10.3389/fmicb.2020.00545 32390953 PMC7193231

[B40] Zhang F. Chen J. Ren W. Lin L. Zhou Y. Zhi X.. (2012). Cloning, expression, and characterization of an alkaline thermostable GH11 xylanase from Thermobifida halotolerans YIM 90462T. J. Industr. Microbiol. Biotechnol. 39, 1109–1116. 10.1007/s10295-012-1119-8 22461083

[B41] Zhang F. Y. He H. H. Deng T. Ge H. R. Yu C. Feng L.. (2020). N-terminal fused signal peptide prompted extracellular production of a bacillus- derived alkaline and thermo stable xylanase in *E. coli* through cell autolysis. Appl. Biochem. Biotechnol. 192, 339–352. 10.1007/s12010-020-03323-9 32382941

[B42] Zhao L. Meng K. Bai Y. Shi P. Huang H. Luo H.. (2013). Two family 11 xylanases from *Achaetomium* sp. Xz-8 with high catalytic efficiency and application potentials in the brewing industry. J. Agric. Food Chem. 61, 6880–6889. 10.1021/jf4001296 23790084

[B43] Zhao Y. Meng K. Luo H. Yang P. Shi P. Huang H.. (2011). Cloning, expression, and characterization of a new Xylanase from alkalophilic *Paenibacillus* sp. 12-11. J. Microbiol. Biotechnol. 21, 861–868. 10.4014/jmb.1102.02024 21876378

