# Supplementary figures and images for: Expression, Characterization and Its Deinking Potential of a Thermostable Xylanase From Planomicrobium glaciei CHR43
Source: Front Bioeng Biotechnol. 2021 Feb 17;9:618979. doi: 10.3389/fbioe.2021.618979 (PMC7928332; doi:10.3389/fbioe.2021.618979)

**Supplement:**

**
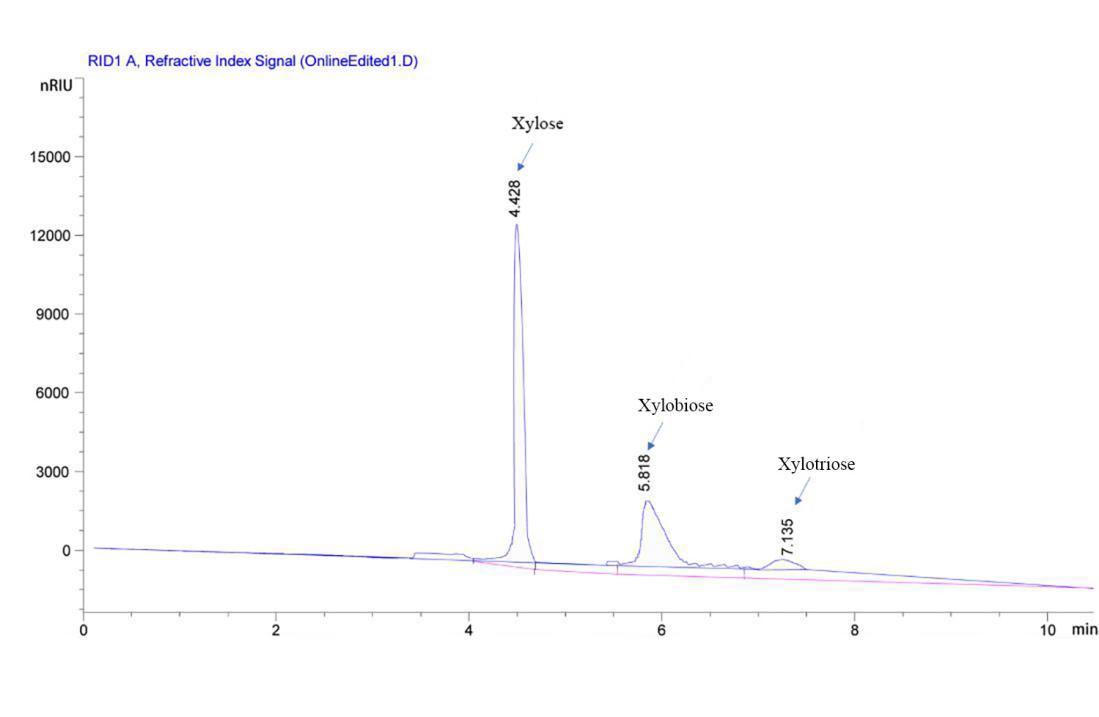
**

**Fig. S1** HPLC analysis of xylan hydrolysis catalyzed by Pg-Xyn

Supplement: Supplementary file 1 [file Data_Sheet_1.DOCX]
